# Formulation and investigation of pilocarpine hydrochloride niosomal gels for the treatment of glaucoma: intraocular pressure measurement in white albino rabbits

**DOI:** 10.1080/10717544.2020.1775726

**Published:** 2020-06-18

**Authors:** Neelam Jain, Anurag Verma, Neeraj Jain

**Affiliations:** a Department of Pharmacy, Oriental College of Pharmacy and Research, Oriental University, Indore, India; b School of Pharmaceutical Sciences, IFTM University, Moradabad, India; c Department of Pharmaceutical Sciences, Dr. Hari Singh Gour University, Sagar, India

**Keywords:** Niosomes, pilocarpine HCl, niosomal gel, Draize test, IOP

## Abstract

The present study was focused on investigating niosomal gels loaded with cholinergic drug; pilocarpine HCl, for prolonged precorneal residence time and improved bioavailability for glaucoma treatment. Pilocarpine HCl niosomes were prepared using various nonionic surfactants (span 20, span 60 and span 80), in the presence of cholesterol in different molar ratios by ether injection method. The selected formulations were incorporated into carbopol 934 and locust bean gum-based gels. TEM analysis confirmed that niosomes formed were spherical in shape and has a definite internal aqueous space with uniform particle size. Formulation F4 composed of span 60 and cholesterol (1:1) gave the highest entrapment (93.26 ± 1.75%) and slower release results after 8 hours (Q8h = 60.35 ± 1.87%) among other formulations. The *in-vitro* drug permeation studies showed that there was a prolonged release of drug from niosomal gels as compared to niosomes itself. Considering the *in-vitro* drug release, niosomal gel formulation G2 was the best among the studied formulations. The release data were fitted to an empirical equation, which indicated that the release follows non-Fickian diffusion mechanism. The stability study revealed that incorporation of niosomes in gel increased their stability than the niosome itself. No signs of redness, inflammation, swelling or increased tear production were observed over the study period for tested formulation by Draize’s test. The intraocular pressure (IOP) lowering activity of G2 formulation showed relative bioavailability 2.64 times more than bioavailability of marketed Pilopine HS® gel. These results suggest that the niosomal gels containing pilocarpine HCl are promising ocular carriers for glaucoma treatment.

## Introduction

Glaucoma is a prevalent neurodegenerative disorder of the eye. Increased intraocular pressure (IOP) and subsequent retinal ganglion cell (RGC) death leading to the loss of visual field characterizes the pathology of primary open angle glaucoma (POAG), which is the most common form. The disease affects over 66 million people worldwide, causing bilateral blindness in 6.8 million (Weinreb & Khaw, 2004; Jain et al., 2019). Patients with POAG typically exhibit increased resistance to the outflow of aqueous humor through the trabecular meshwork, which can result in an increase in IOP and subsequent cell death from compression of the optic nerve axons (Margalit & Sadda, 2003). However, IOP is the primary risk factor causing the loss of RGCs, the strategies of treatment mostly involve lowering IOP (Brubaker, 2003). Urrent treatment options primarily aim at decreasing IOP by utilizing pharmacological agents, laser therapy and surgery. The method of reducing IOP is by enhancing the outflow of humor from the eyes through the use of muscarinic acetylcholine receptor agonists (Woodward & Gil, 2004; Schwartz & Budenz, 2004).

Pilocarpine hydrochloride is a drug used in the treatment of chronic open-angle glaucoma for over 100 years (Rosin, 1991). It is a parasympathomimetic alkaloid obtained from the leaves of tropical South American shrubs from the genus *Pilocarpus*. It is a nonselective muscarinic receptor agonist which acts therapeutically at the muscarinic acetylcholine receptor M_3_, found on the iris sphincter muscle, causing the muscle to contract resulting in pupil constriction (miosis). Pilocarpine hydrochloride also acts on the ciliary muscle and causes it to contract. When the ciliary muscle contracts, it opens the trabecular meshwork through increased tension on the scleral spur. This action facilitates the rate that aqueous humor leaves the eye to decrease in IOP (Khaw et al., 2004). The major drawbacks associated with pilocarpine HCl, administered as an eye drop, was its low ocular bioavailability (1–3%) and short precorneal residence time. These problems can be minimized by the use of niosomal vesicular system.

Niosomes are formed from the self-assembly of nonionic amphiphiles in aqueous media resulting in closed bilayer structures (Uchegbu & Vyas, 1998), which can entrap both hydrophilic and lipophilic drugs either in an aqueous layer or in vesicular membrane (Carafa et al., 1998). Niosomes in topical ocular delivery are preferred over other vesicular systems because of the chemical stability; low toxicity due to their nonionic nature; handling surfactants with no special precautions or conditions; the ability to improve the performance of the drug via better availability and controlled delivery at a particular site and being biodegradable, biocompatible and non-immunogenic (Carafa et al., 2002). Some researchers reported that there was approximately a 2.5 times increase in the ocular bioavailability of timolol maleate (a water soluble drug) encapsulated in niosomes as compared to timolol maleate solution (Vyas et al., 1998).

Niosomes, administered as an ophthalmic gel, are capable of localizing and maintaining drug activity at its site of action due to their easy transition through ocular barrier with reduced drug frequency and toxicity. Moreover, niosomes-based ocular gel containing bioadhesive polymer helps the drug to remain adhered to corneal surface for a long period of time. Hence, precorneal residence time is increased, resulting in significant enhancement of ocular bioavailability.

Therefore, the present study aims to develop and evaluate niosomal gel formulations containing pilocarpine HCl to achieve prolonged precorneal residence time and improved bioavailability. Niosomes were prepared using various nonionic surfactants (span 20, span 60 and span 80) in the presence of cholesterol in different molar ratios by ether injection method. Selected formulations of niosomes were incorporated into carbopol 934 (1% w/w) and locust bean gum (3% w/w) gels.

## Materials and methods

The pilocarpine HCl was kindly received as a gift sample by M/s Zydus Cadila Health Care Ltd. (Ahmedabad, India). Sorbitan monolaurate (span 20), sorbitan monosterate (span 60), sorbitan monooleate (span 80), cholesterol, locust bean gum and carbopol 934 were procured from Loba Chemie Pvt. Ltd. (Mumbai, India). Isopropanol, methanol, acetone, chloroform, boric acid, sodium hydroxide, sodium bicarbonate, potassium chloride, glacial acetic acid, magnesium, sodium chloride, calcium chloride dehydrate, potassium dihydrogen phosphate and disodium hydrogen phosphate were purchased from were purchased from S.D Fine chemicals (Mumbai, India). Double distilled water was used throughout the study.

### Preparation of pilocarpine hydrochloride–loaded niosomes

Pilocarpine hydrochloride–loaded niosomes were prepared by ether injection technique using nonionic surfactants (span 20, span 60 and span 80) and cholesterol in different ratios (Jain et al., 2019) as shown in Table 1. For each ratio, nonionic surfactant and cholesterol were weighed accurately and dissolved in 20 ml of diethyl ether. Pilocarpine hydrochloride (40 mg) was then dissolved in this lipid solution. The resulting solution was taken in a syringe and injected slowly through a 16 gauge needle into 10 ml of aqueous phase (phosphate buffer saline pH 7.4) held in a beaker maintained at 60 °C to 65 °C and agitated slowly. Vaporization of diethyl ether resulted in the formation of niosomes. The prepared niosomes were separated by ultracentrifugation (Remi C-24, Mumbai, India) at 4 °C.

**Table 1.** Composition for niosomes.

Formulation Code	Surfactant	Amount of surfactant (mg)	Cholesterol (mg)	Drug (mg)	Ratio (Surfactant: Cholesterol)
F1	Span 20	100	100	40	1:1
F2	Span 20	200	100	40	2:1
F3	Span 20	100	200	40	1:2
F4	Span 60	100	100	40	1:1
F5	Span 60	200	100	40	2:1
F6	Span 60	100	200	40	1:2
F7	Span 80	100	100	40	1:1
F8	Span 80	200	100	40	2:1
F9	Span 80	100	200	40	1:2

### Evaluation of pilocarpine hydrochloride loaded niosomes

#### Drug entrapment efficiency (% EE)

The proportion of encapsulated pilocarpine hydrochloride was obtained by ultracentrifugating 1 ml of the niosomal suspension at 15,000 rpm for 1 hr using a cooling centrifuge at 4 °C (Remi C-24, Mumbai, India). The niosomes were separated from the supernatant and were washed twice, each time with 1 ml phosphate buffered saline, and recentrifuged again for 1 hr. The amount of entrapped pilocarpine hydrochloride was determined by lysis of the separated vesicles with isopropanol. A 100 μl sample of niosomes was mixed with 1 ml of isopropanol; the volume was completed to 10 ml with phosphate buffered saline and covered with parafilm to prevent evaporation. The concentration of the drug was determined by UV spectrophotometer (UV 1700 Pharm Spec, Shimadzu, Japan) at 215 nm. Study was done in triplicate and % drug entrapment efficiency can be calculated by using following formula:

$$\text{\% EE}=\frac{\text{Actual drug content}}{\text{Theoretical drug content}}\text{ × }100$$

### Vesicle size and zeta potential measurements

Vesicle size of different niosomal formulations were observed under an optical microscope (Olympus Model BX 41, Japan) at suitable magnification. The measurements were done in triplicate and vesicle size was recorded. The zeta potential of the prepared niosomal formulations was determined by Zetasizer Nano ZS-90 (Malvern Instruments Ltd., UK) using 0.1 M KCl buffer in demineralized water at 25 °C (Levchenko, 2002).

### Transmission electron microscopy (TEM)

The prepared niosomal formulations were characterized for their shape using transmission electron microscope (JEM-200 CX, JEOL, Tokyo, Japan) at 80 KV, after being stained and TEM micrograph was taken at suitable magnification (Hu & Rhodes, 1999).

### Differential scanning calorimetric (DSC) study

Differential scanning calorimetric analysis were performed on the pure drug (pilocarpine HCl) and drug loaded niosomes. Initially, the moisture was removed by heating the samples and then, each sample (about 3-7 mg) was accurately weighed into platinum crucible 40 μl aluminum pan in hermetically sealed condition, where alpha alumina powder used as a reference. Thermogram was recorded from 50 °C to 300 °C at the heating rate of 20 °C/min under a constant flow of an inert nitrogen gas atmosphere with the flow rate of 20 ml/min. These analyses were done on Perkin-Elmer instrument (Pyris-1, Osaka, Japan) available at Department of Textile Technology, Indian Institute of Technology, New Delhi, India.

### *In vitro* drug release from niosomes

The *in vitro* release of entrapped drug within niosomes was determined using membrane diffusion technique. The niosomal formulation equivalent to 4 mg of pilocarpine HCl was placed in a glass tube that was previously covered with presoaked cellulose membrane, which acts as a donor compartment. The glass tube was placed in a beaker containing 50 ml of simulated lachrymal fluid (pH 7.4), which acted as receptor compartment. The whole assembly was fixed in such a way that the lower end of the tube containing suspension was just touching (1-2 mm deep) the surface of diffusion medium. The temperature of receptor medium was maintained at 37 ± 1 °C and agitated at 100 rpm speed using magnetic stirrer. Aliquots of 5 ml sample were withdrawn periodically and after each withdrawal same volume of medium was replaced. The collected samples were analyzed spectrophotometrically at 215 nm using simulated lachrymal fluid (pH 7.4) as blank.

### Preparation of gels containing pilocarpine hydrochloride niosomes

Selected drug loaded niosomes (equivalent to 0.4% w/w drug) were incorporated into different gel bases as shown in Table 2. The polymers used were locust bean gum and carbopol 934. The required quantity of these polymers was weighed and dispersed in a small amount of phosphate buffer saline pH 7.4 to prepare an aqueous dispersion and sterile in hot air oven at 160 °C for 1 hr. The aqueous dispersion was allowed to hydrate for 4–5 hrs. The pH was adjusted to 6 by addition of triethanolamine solution. The final weight of the gel was adjusted with phosphate buffer saline pH 7.4. Niosomal suspension containing drug was separated from aqueous medium by ultracentrifugation at 15000 rpm at 4 °C and was added gently by vortex in the sterile blank gel under laminar air flow cabinet. The solution was made isotonic with sodium chloride (0.9% w/v). Then, benzalkonium chloride (0.001% v/v) was added as a preservative. The gel was made consistent with glycerin (10% w/v). Vortexing was continued until a homogenous niosomal gel was obtained and the gel was then sonicated to become bubble-free. The prepared gels were filled in amber colored glass vials refrigerated at 4 to 8 °C.

**Table 2.** Composition for niosomal gels.

Gel formulation	Niosomes loaded	Locust bean gum (% w/w)	Carbopol 934 (% w/w)
G1	F1	3%	1%
G2	F4	3%	1%
G3	F7	3%	1%

### Evaluation of gels containing pilocarpine hydrochloride niosomes

#### Rheological studies

The viscosity of different gel formulations using Brookfield DV-II + Pro model LV viscometer equipped with a helipath stand and T bar spindles. Viscosity measurements were made at variable shear rate. It was carried out at constant temperature of 37 ± 1 °C, but varying the rotation speed of the spindle from 10 to 100 rpm in a small sample adaptor. Evaluations were done in triplicate.

#### Spreadability

The therapeutic efficacy of a formulation also depends on its spreading value. Spreadability is expressed in terms of time in seconds taken by two slides to slip off from the formulation, placed in between, under the application of a certain load. Lesser the time is taken for the separation of the two, better the spreadability. Two glass slides of standard dimensions were selected. 1 g gel was placed over one of the slides. The other slide was placed on top of the formulations and was sandwiched between the two slides across the length of 5 cm along the slide. 100 g weight was placed upon the upper slide so that the formulation between the two slides was pressed uniformly to form a thin layer. The weight was removed and the excess of formulation adhering to the slides was scrapped off. One of the slide was fixed on which the formulation was placed. The second movable slide was placed over it, with one end tied to a string to which load could be applied by the help a simple pulley and a pan. A 30 g weight was put on the pan and the time taken for the upper slide to travel the distance of 5.0 cm and separate away from the lower slide under the direction of the weight was noted. Spreadability was then calculated by using the formula: (Shalin et al., 2017)

S=M.L/T

where S = Spreadability, M = Weight tide to upper slide (gm), L = Length of glass slide (cm), T = Time taken to separate the slide completely from each other (sec).

### *Ex-vivo* bioadhesive strength

Freshly excised conjunctiva of an adult goat was used as model membrane for the measurement of isotonic phosphate buffer, pH 7.4 before use. Bioadhesive strength of ocular gel was measured on a modified two-arm physical balance. The pan at the left arm of the balance was detached and a vertical thread was hung to the lever of the left arm which had a rubber stopper tied to its end, hanging downward. The formulation to be tested was adhered to the downward facing side of the rubber stopper. Conjunctival membrane was tied onto the open mouth of a glass vial which was filled with isotonic phosphate buffer. The vial was fitted in the center of a glass beaker filled with simulated tear fluid (pH 7.4) maintained at 37 °C. The apparatus was set such that the vial (conjunctival membrane tied on it, facing upward) lies exactly below the rubber stopper (insert tied on it, facing downward). The rubber stopper was lowered so as to make the formulation come in contact with the membrane. After facilitating the contact between the two, weight was put on the right limb of balance (gram force) required to detach the formulation from the conjunctival surface (Sultana et al., 2006). The detachment stress (dynes/cm^2^) was then calculated by using formula:

Detachment stress=mg/A

where m = Weight required for detachment (gm), g = Acceleration due to gravity (980 cm/s^2^), A = Area of mucosa exposed (cm^2^).

### *In vitro* drug permeation from niosomal gels

The *in vitro* drug permeation of niosomal gels was studied through cellophane membrane using a diffusion cell (Kurniawansyah et al., 2018), as in case of niosomal formulations.

## Release kinetics modeling

In order to investigate the release mechanism of drug from niosomal gel preparations, the *in-vitro* release data were fitted with the following mathematical models: (Costa & Lobo, 2001).

Zero-order kinetics equation:

$$Q_{t}=k_{0}.t$$

where Q_t_ is the amount of drug released at time t, k_0_ is the zero-order release rate constant, t is the time.

First-order kinetics equation:

$${\;\text{ln}\;\;Q}_{t}={\;\text{ln}\;\;Q}_{0}\;–{\;k}_{1.}t$$

where Q_t_ is the amount of drug released at time t, Q_0_ is the initial amount of drug in the solution, k_1_ is the first-order release rate constant.

Higuchi model kinetics equation: (Higuchi, 1963)

$$Q_{t}={\;k}_{H}.{\;t}_{1/2}$$

where Q_t_ is the amount of drug released at time t, k_H_ is the Higuchi release rate constant.

Korsmeyer–Peppas model kinetics equation: (Ritger & Peppas, 1987)

$$M_{t}/M_{\infty }={\;K}_{\text{KP}}.t^{n}$$

where M_t_ is the fraction of drug released at time t, M_∞_ is the fraction of drug released at infinite time, K_KP_ is the Korsmeyer–Peppas release rate constant, n is the release exponent. The value of exponent (*n*) indicates the mechanism of drug release.

### Stability studies

Adequate samples of each of the selected niosomal formulations (niosome and niosomal gel) from formulation F4 and G2 were sealed into 10 ml ambered glass vials and stored at temperature 4 ± 1 °C in a refrigerator and 37 ± 1 °C in a thermostat controlled hot air oven for 28 days. After every 7 days the formulations were evaluated for % pilocarpine HCl retained in gel formulation. The initial drug content was considered as 100% (Sawant et al., 2016).

### *Ex vivo* drug permeation study

Freshly excised whole cow eyeball was procured from a slaughter house and transported to the laboratory in cold condition. They were maintained in normal saline at 4 °C. The cornea was then carefully removed along with a 5–6 mm of surrounding scleral tissue and washed with cold saline. The washed cornea was preserved in freshly prepared phosphate buffer (pH 7.4) and stored under refrigeration until the time of the study. The preserved cornea was mounted on Franz diffusion cell by sandwiching between the donor and receptor compartment. It was positioned on the donor half-cell such that the epithelial surface was facing the donor solution. The receptor half-cell was positioned symmetrically opposing the donor half-cell. The half-cells were secured together with a clamp. This procedure prevents any leaks (Rasool et al., 2014).

One gram of selected niosomal gel formulation (G2) was placed inside the donor half-cell over the corneal membrane. The entire surface of the cornea was in contact with the receptor compartment that contained 50 ml of simulated tear fluid (pH 7.4), which was stirred continuously using a magnetic stirrer at 100 rpm to simulate blinking action. At predetermined time intervals for up to 8 hr, 5 ml aliquots of the release medium were withdrawn for analysis and were replaced with equal volume of release medium at the same temperature to maintain constant volume. *Ex-vivo* drug permeation through cornea from niosomal gel was analyzed spectrophotometrically using UV-spectrophotometer at 215 nm and compared with marketed pilocarpine formulation (Pilopine HS® gel). Results were tabulated and graph was plotted as cumulative percentage of drug permeated versus time for niosomal gel formulation (G2). Study was done in triplicate.

### Ocular irritancy test (Draize’s test)

Rabbits were divided into two groups (four rabbits in each group). G2 niosomal gel formulation and marketed Pilopine HS® gel were applied to Group I and Group II of rabbit’s eyes respectively. The untreated eye serves as control. 20 μl of the representative formulation was instilled into the lower conjunctival sac of the rabbit’s right eye, while the left was kept as a control. The solutions were instilled periodically twice a day. The test eye were examined for any abnormality (irritation signs) that were recorded before treatment and 30 min, 1 h, 24 h, 48 h, 72 h, 7 days, 14 days and 21 days after treatment (Morsi et al., 2016). The common irritation signs are expected to be conjunctival redness, swelling and discharge scoring 0 (absence) to 4 (highest).

#### *In vivo* antiglaucoma activity by measurement of intraocular pressure (IOP)

Rabbits were randomly divided into three groups (six rabbits in each group). Group I served as control while Group II and Group III were treated with G2 niosomal gel formulation and marketed Pilopine HS® gel respectively (Preethi & Kunal, 2016; Erfani et al., 2013). Glaucoma disease was induced by Bonomi et al., 1978 method. Rabbits were treated with subconjunctival injections of 0.25 ml Betamethasone injection (Betamethasone sodium 4 mg/ml) every week for three successive weeks in left and right eyes. Local anesthetic eye drops (Benox®) were used prior to subconjunctival injection. The activity was confirmed by noticing a bulge formation at the site of injection. The right eye of each rabbit was kept as control and the left eye was treated for glaucoma using 40 µl (equivalent to 40 µg of pilocarpine HCl) of selected formulation (G2) and marketed Pilopine HS® gel for group II and group III, respectively. The intraocular pressure (IOP) readings were measured using Schiotz Tonometer, before drug administration and 2 h, 4 h, 6 h, 7 h, 8 h, 9 h, 10 h, 11 h and 24 h after drug administration. IOP was measured three times at each time interval and the means were recorded.

The change in IOP (ΔIOP) is expressed as follows:

ΔIOP=IOP dosed eye – IOP control

The pharmacokinetic parameters taken into consideration such as the maximum percentage decrease in IOP (% IOP_max_) and the time of maximum response (T_max_) were estimated through constructing % ΔIOP versus time curves.

$$\text{\% Maximum reduction of IOP }(\text{\% IOPmax})=\frac{\mathrm{IOPo}-\mathrm{IOPmax}}{\mathrm{IOPo}}\text{ × }100$$

where IOP_o_ is the intra-ocular pressure at 0 time, IOP_max_ is the intra-ocular pressure at T_max._

The area under the curve (AUC) was calculated according to trapezoidal rule. Relative bioavailability for formulations was determined through the equation below:

$$\text{Relative bioavailability}=\frac{\mathrm{AUCt}}{\mathrm{AUCs}}$$

where AUCt and AUCs are the AUC estimated for test formulation and standard formulation (marketed Pilopine HS® gel).

## Results and discussion

### Evaluation of pilocarpine hydrochloride-loaded niosomes

#### Drug entrapment efficiency in niosomes

##### Effect of surfactant type

From the results in Table 3, It was observed that the entrapment efficiency of niosomes composed of span 60 were superior as compared to those prepared from span 20. The formulation containing span 80 showed the lowest entrapment efficiency. This can be due to:

**Table 3.** Evaluation of niosomes.

Formulation Code	Entrapment efficiency (%)	Vesicle size (µm)	Zeta potential (mV)	Q8h (%)
F1	81.43 ± 2.09	1.44 ± 2.76	−31.04 ± 0.25	78.81 ± 4.82
F2	86.17 ± 3.07	1.56 ± 1.59	−29.54 ± 0.72	73.15 ± 6.32
F3	78.56 ± 0.99	2.71 ± 0.57	−28.84 ± 0.27	68.74 ± 0.78
F4	93.26 ± 1.75	6.13 ± 0.31	−15.04 ± 0.45	60.35 ± 1.87
F5	90.66 ± 3.80	5.16 ± 0.27	−22.21 ± 1.28	63.54 ± 4.42
F6	84.06 ± 9.36	7.12 ± 2.80	−24.64 ± 0.53	66.98 ± 3.83
F7	79.11 ± 3.96	3.68 ± 0.69	−30.04 ± 0.72	74.04 ± 2.25
F8	83.47 ± 2.65	3.09 ± 1.32	−28.84 ± 0.47	75.33 ± 0.89
F9	72.33 ± 2.03	4.24 ± 0.41	−27.32 ± 0.34	71.05 ± 0.93

- The hydration temperature used to make niosomes should usually be above the gel to liquid phase transition temperature of the system that results in niosomes that are less leaky and have high entrapment efficiency. Span 60 has highest phase transition temperature (50 °C) as compared to span 20 (16 °C) and span 80 (−12 °C) and hence high entrapment efficiency.
- The length of alkyl chain of surfactant has a prominent effect on permeability of prepared niosomes. As the length of surfactant increases, entrapment efficiency also increases. Span 60 has a longer saturated alkyl chain (C18) compared to span 20 (C12), so it produces niosomes with higher entrapment efficiency. Span 60 and span 80 have the same head group but span 80 has an unsaturated alkyl chain which results in enhanced permeability and decreased entrapment.

### Effect of cholesterol weight ratio

The entrapment efficiency decreased with increasing cholesterol concentration for span 60 formulations. This may be due to higher amounts of cholesterol competing with the drug for packing space within the bilayer, hence excluding the drug as the amphiphiles assemble into vesicles. Another explanation may be that the increasing cholesterol beyond a certain concentration can disrupt the regular linear structure of the vesicular membranes (Bayindir & Yuksel, 2010). Formulation F4 shows the maximum % EE among all the formulations.

### Vesicle size and zeta potential measurements

The size of particles in ophthalmic dosage forms plays an important role in the irritation potential of formulation; hence, it is recommended that the particles of ophthalmic solution should be less than 10 µ to minimize irritation to the eye (Patel et al., 2012). Vesicle size of all formulations was ranges between 1.44 ± 2.76 µm to 7.12 ± 2.80 µm as shown in Table 3. These sizes are well acceptable for ocular administration. From Table 3, it was observed that the niosomes prepared using span 60 is larger in size than those prepared using span 20 and span 80. Span 60 has a longer saturated alkyl chain and it was reported that surfactants with longer alkyl chains generally give larger vesicles.

**Figure 1.**
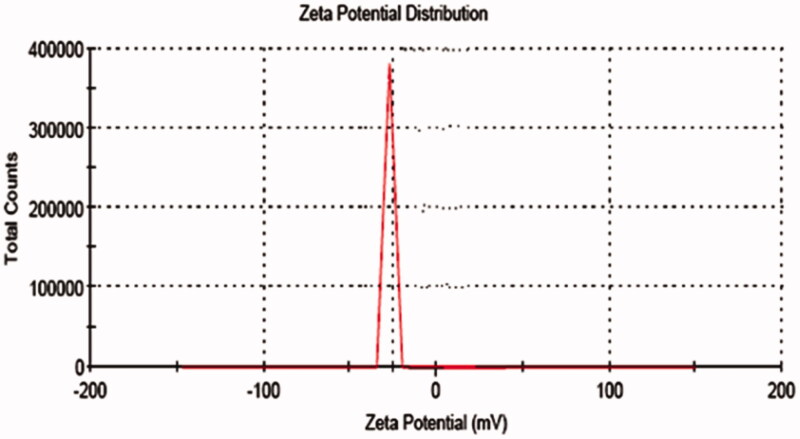
Zeta potential of niosomal formulation.

The zeta values for niosomal formulations were found to be in range of −15.04 ± 0.45 mV to −31.04 ± 0.25 mV as shown in Table 3. The zeta potential of the niosome under study was found to be −31.04 ± 0.25 mV as shown in Figure 1. The results revealed that the zeta values of the vesicles increase toward negative with increasing the HLB values of the surfactants. The effect of HLB values of surfactants on zeta potential could be explained in terms of surface energy, which tends to increase with increase in HLB values toward the hydrophilicity. Increase in surface energy of the vesicles leads to increase the values of zeta potential toward negative (Kamboj et al., 2014). The high negative surface charge on niosomes indicates higher stability because of the anticipated surface repulsion between similarly charged particles therefore, inhibiting aggregation of the colloidal niosomal particles (Satish et al., 2016). It was observed that all the formulations were sufficient to keep the particles stable.

### Transmission electron microscopy

**Figure 2.**
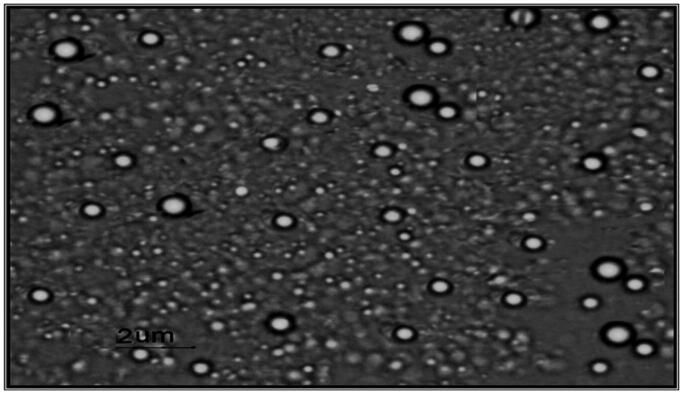
Transmission electron micrograph of niosomal formulation at 30000X.

Negative stain transmission electron micrographs of selected pilocarpine HCl loaded niosomal formulation was shown in Figure 2. It was demonstrated that the vesicles are well identified and present in a nearly perfect sphere like shape with a smooth surface and having a definite internal aqueous space.

### Differential scanning calorimetric (DSC) study

**Figure 3.**
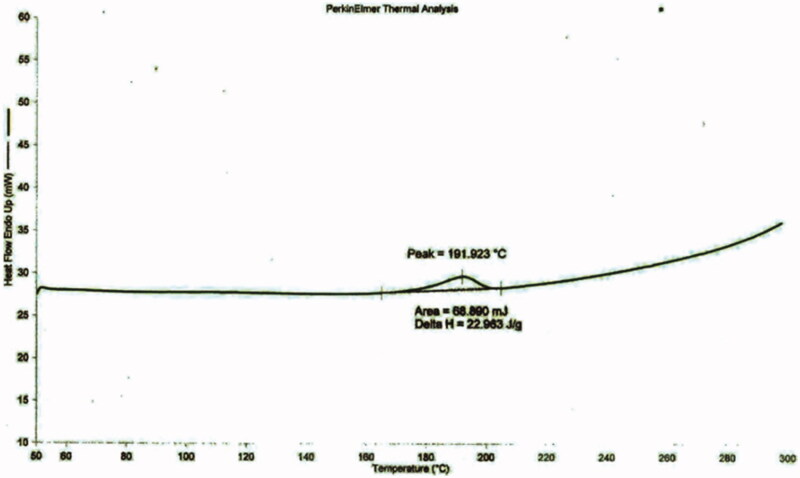
DSC thermogram tracings of pure drug.

**Figure 4.**
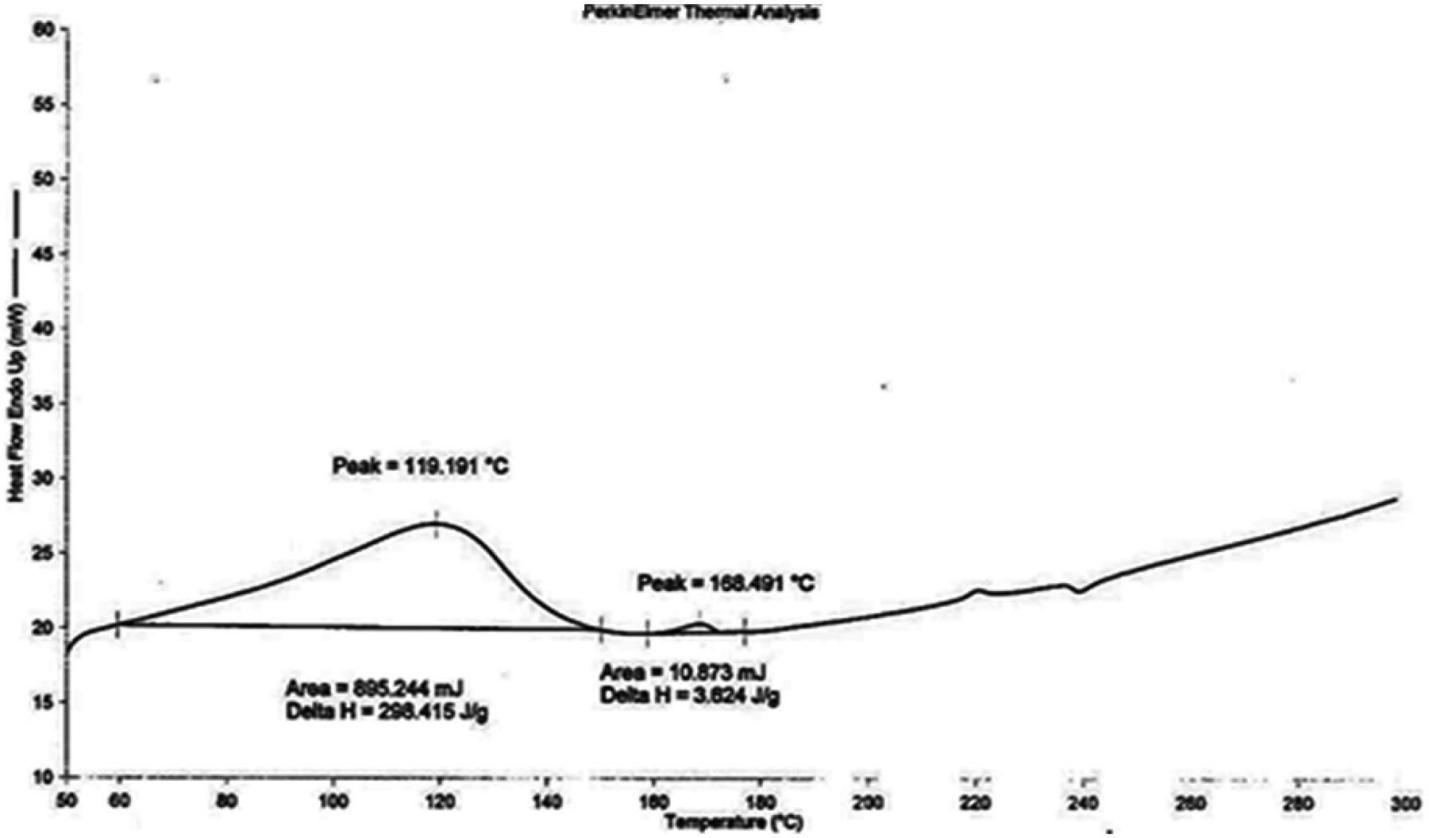
DSC thermogram tracings of drug loaded niosomes.

DSC thermogram of pure drug (pilocarpine HCl) and drug loaded niosomes were displayed in Figures 3 and 4, respectively. DSC thermogram of pure pilocarpine HCl showed a sharp exothermic peak at 191.923 °C (area = 68.890 mJ, delta H = 22.963 J/g) indicating the crystal melting point of the drug. DSC thermogram of drug-loaded niosomes exhibited endotherms at 119.191 °C (area = 895.244 mJ, delta H = 298.415 J/g) and 168.491 °C (area = 10.873 mJ, delta H = 3.624 J/g). Endotherm at 119.191 °C corresponds to the thermal decomposition of the surfactant (span 60), while endotherm at 168.491 °C indicated the increase in phase transition temperature of niosomes upon loading with drug. Absence of the melting endotherm of pilocarpine HCl suggested that drug changed from crystalline to amorphous state. These results suggest significant interaction of drug with the bilayer structure and can account for the enhanced entrapment of drug into niosomal formulations and sustained drug release (Nagarsenker et al., 1999).

### *In-vitro* drug release from niosomes

**Figure 5.**
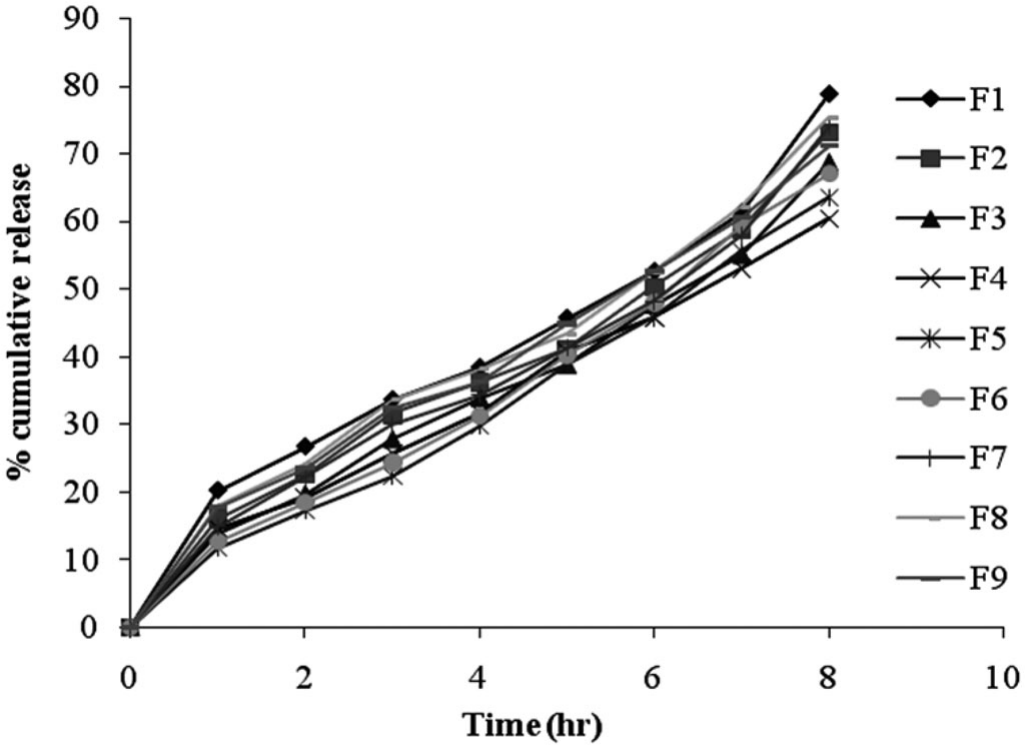
*In vitro* release profile of different niosomal formulations.

The percentage of drug released after 8 hr from the prepared niosomal vesicles at simulated lachrymal fluid of pH 7.4 varied from 60.35 ± 3.83% to 78.81 ± 4.82% as shown in Figure 5 and the data was presented in Table 3. By inspection of the data, it could be concluded that niosomal formulations prepared using span 60 (1:1) yielded a lower rate of release compared to span 20 and span 80 niosomes. This can be explained by the fact that niosomes exhibit an alkyl chain length dependent release and the higher the chain length, the lower the release rate (Devaraj et al., 2002). By reviewing the data, it has been revealed that release after 8 hours for the niosomal formulations can be arranged in the following decreasing order: F1 > F8 > F7 > F2 > F9 > F3 > F6 > F5 > F4. From results, it is obvious that the increase of cholesterol molar ratio reduced the efflux of the drug from niosomal preparations, which is in accordance with its membrane-stabilizing ability. Cholesterol is known to abolish the gel to liquid-phase transition of niosome systems, resulting in niosomes that are less leaky. Therefore, the diffusion of pilocarpine HCl entrapped in the hydrophobic regions of the vesicles would be expected to occur over a prolonged period of time (Ruckmani et al., 2000).

### Evaluation of gels containing pilocarpine hydrochloride niosomes

#### Rheological studies

**Figure 6.**
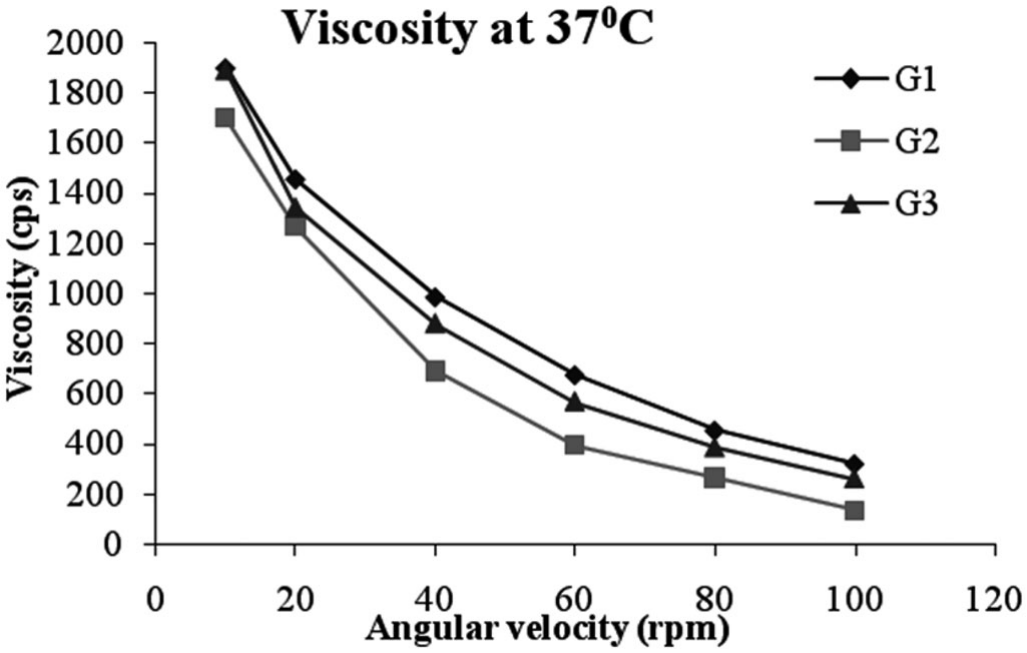
Viscosity of niosomal gel formulations.

The viscosity of the all gel formulations ranged from 135-1900 cps as shown in Table 4. A shear thinning formulation with a high viscosity at low shear rate and lower viscosity at higher shear rates will be preferable. All the formulations showed pseudoplastic rheological flow, as evidenced by shear thinning and increase in shear stress with increased angular velocity. From Figure 6, it was observed that viscosity of all the formulations was decreasing with the increase in shear rate. The non-Newtonian formulations with pseudoplastic properties can acquire a viscosity decrease with increasing shear rate, creating blinking and ocular movement. Pseudoplasticity is thus interesting because it offers significantly less resistance to blinking and shows much greater acceptance than viscous Newtonian formulation (Chaudhari & Desai, 2019).

**Table 4.** Viscosity of the niosomal gel formulations (Mean ± SD, *n* = 3).

Code	Viscosity in cps					
	10 rpm	20 rpm	40 rpm	60 rpm	80 rpm	100 rpm
G1	1900 ± 1.70	1457 ± 0.40	986 ± 0.05	675 ± 0.03	453 ± 0.01	320 ± 0.03
G2	1700 ± 0.05	1267 ± 0.60	689 ± 0.20	394 ± 0.10	264 ± 0.05	135 ± 0.03
G3	1890 ± 0.68	1342 ± 0.68	880 ± 0.34	568 ± 0.12	385 ± 0.08	260 ± 0.05

#### Spreadability

The spreadability of the prepared niosomal gel (G1, G2 and G3) was ranges from 1.41 ± 0.72 to 2.35 ± 0.25 g.cm/s as shown in Table 5. It was observed that with increase in polymer concentration, the spreadability decreased due to the increase in viscosity of the formulation (Shilakari et al., 2016). Formulation G2 has higher spreadability (2.35 ± 0.25 g.cm/s) with low viscosity as compared to formulation G1 and G3. One of the criteria for a gel to meet the ideal quality is that it should possess good spreadability. It is the term expressed to denote the extent of area, to which gel readily spreads on application site. Lesser the time is taken for separation of two slides, better the spreadability.

**Table 5.** Some characteristics of niosomal gels.

Gel formulation	Spreadability (g.cm/s)	Bioadhesive strength (dynes/cm^2^)	Q8h (%)
G1	1.41 ± 0.72	2036 ± 0.16	62.89 ± 2.21
G2	2.35 ± 0.25	2564 ± 0.39	50.13 ± 0.81
G3	2.16 ± 0.27	1968 ± 0.23	56.94 ± 1.48

#### *Ex-vivo* bioadhesive strength

The bioadhesive strength of the niosomal gel formulations was ranges from 1968 ± 0.23 to 2564 ± 0.39 dynes/cm^2^ respectively as shown in Table 5. The bioadhesive values show considerable potential of sustaining the residence and enhancing contact with ocular tissue. Formulation G3 showed least bioadhesive detachment force (1968 ± 0.23 dynes/cm^2^) as compared to G1 (2036 ± 0.16 dynes/cm^2^). The highest bioadhesive detachment force of formulation G2 (2564 ± 0.39 dynes/cm^2^) could be attributed to the fact that an anionic polymer carbopol 934 is a polyacrylic acid derivative. Its mucoadhesive property is due to hydrogen bonding with mucin, resulting in good adhesion (Ludwig, 2005). The adhesive behavior of locust bean gum is due to increased viscosity in polymer solution, resulting in effective bioadhesion.

#### *In vitro* drug permeation from niosomal gels

**Figure 7.**
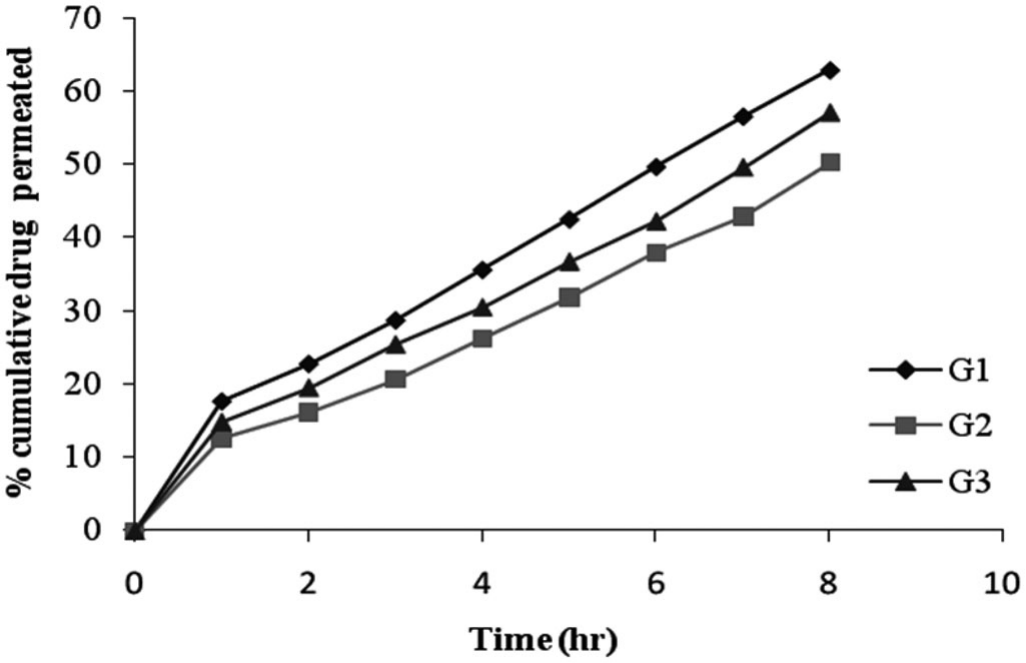
*In vitro* drug permeation of niosomal gels.

**Figure 8.**
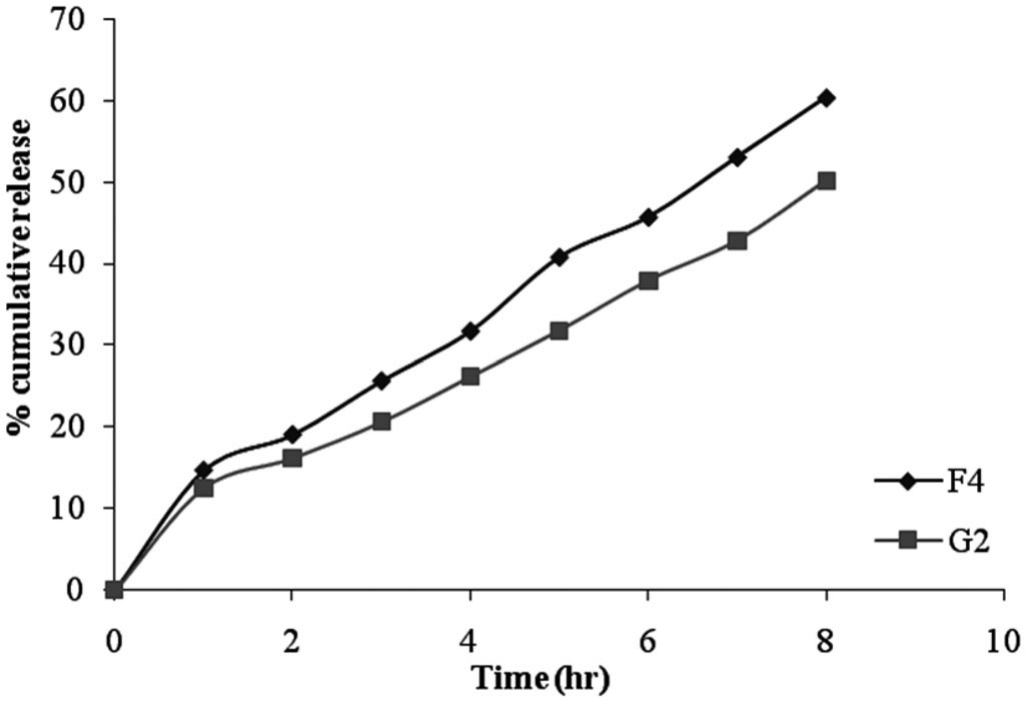
Comparative study for *in-vitro* drug release from niosome (F4) and niosomal gel (G2).

The cumulative percentage of drug permeated from niosomal gel formulation was ranges between 50.13 ± 0.81% to 62.89 ± 2.21% after 8 hrs as shown in Figure 7 and the data was presented in Table 5. The *in vitro* release data of gel formulation G2 shows a lower percentage of drug release as compared with niosomal formulation F4 (Figure 8). These results can be attributed to the presence of bioadhesive polymer which retains the formulation in contact with the eye for a long period of time. Among all the formulations, G2 showed slower drug release (50.13 ± 0.81%) due to high gelling capacity.

## Release kinetics modeling

Kinetics for drug release was studied for zero order kinetics, first order kinetics, Higuchi’s model kinetics and Korsmeyer–Peppas model kinetics with interpretation of diffusional release mechanism and the results were shown in Tables 6 and 7. The determination of the co-relation coefficient (*R*^2^) value indicated that drug release have followed zero order kinetics in case of gel formulation G2 and Korsmeyer-Peppas kinetics in case of formulation G1 and G3 which predicts the release may be diffusion controlled mechanism from the niosomal formulations. The ‘*n*’ value could be used to characterize different drug transport mechanisms and were in the range of 0.5851 to 0.7234 (0.5<*n* < 1.0). This indicates that the release of gel formulations follows non-Fickian diffusion transport mechanism.

**Table 6.** Release kinetics of niosomal gels.

	Zero order		First order		Higuchi		Korsmeyer-Peppas	
Gel Formulation	R^2^	K_0_	R^2^	K_1_	R^2^	K_H_	R^2^	n
G1	0.980	7.262	0.980	0.049	0.965	21.96	0.974	0.58
G2	0.987	5.764	0.977	0.034	0.944	17.18	0.986	0.67
G3	0.983	6.471	0.972	0.041	0.954	19.43	0.985	0.72

**Table 7.** Interpretation of diffusional release mechanisms.

Diffusion exponent (n)	Drug transport mechanism
0.5	Fickian diffusion
0.5<*n* < 1.0	Anomalous (non-Fickian) diffusion
1.0	Case-II transport
*n* > 1.0	Super Case-II transport

### Stability studies

**Figure 9.**
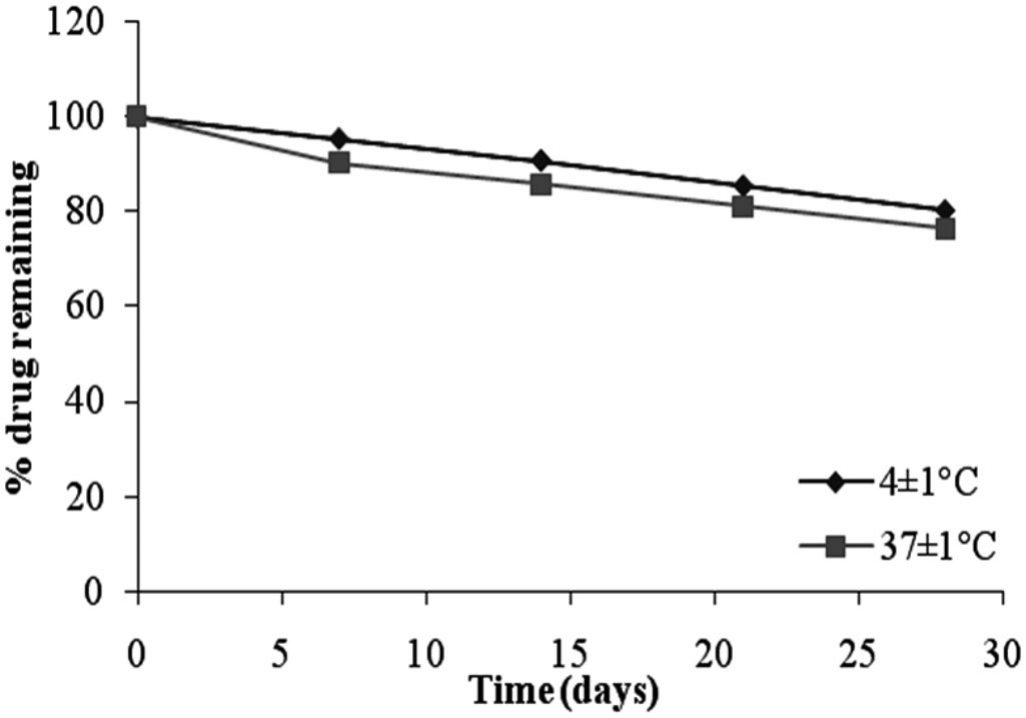
Stability studies of niosome formulation (F4) at different temperatures.

**Figure 10.**
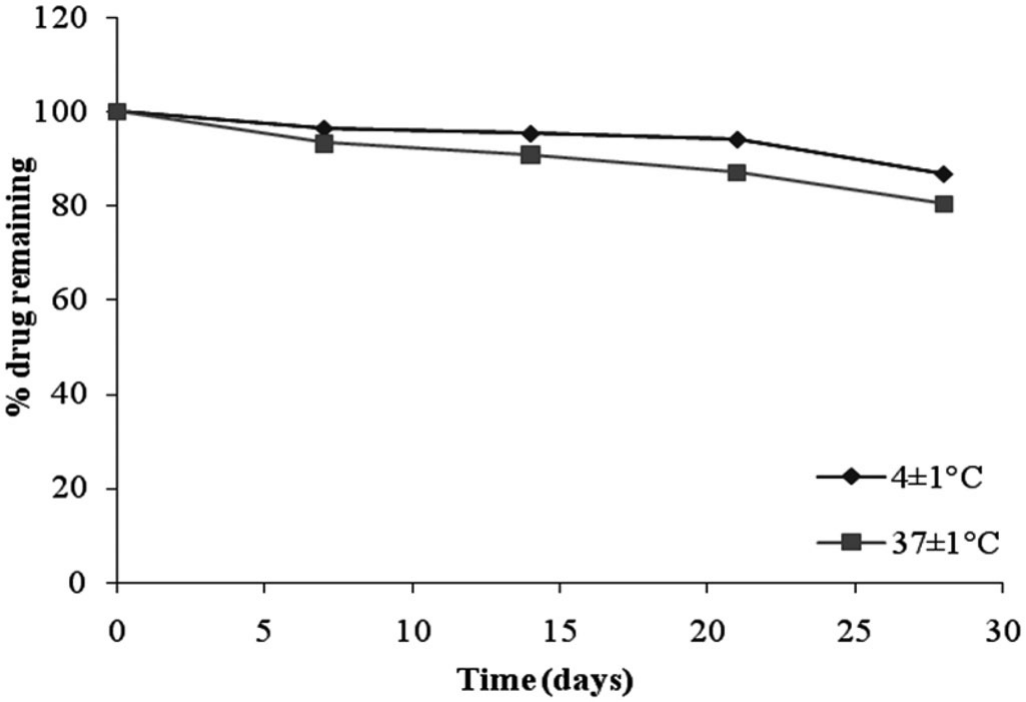
Stability studies of niosomal gel formulation (G2) at different temperatures.

The stability studies revealed that the selected formulation (F4 and G2) met the pharmacopeial requirements of drug content (80–110%) as shown in Figures 9 and 10, respectively. From Table 8, it was observed that at 4 ± 1 °C, the % of drug retained in G2 was 86.76 ± 1.25% and at 37 ± 1 °C, the % remained was 80.58 ± 1.07%. The % pilocarpine HCl retained in F4 was 80.36 ± 1.05% at 4 ± 1 °C and 76.45 ± 2.67% at 37 ± 1 °C. From these results it was concluded that the incorporation of niosomes in gel increased their stability than the niosome itself. This may be due to the rigidity of gel structure which resists the leakage of drug.

**Table 8.** Percent pilocarpine HCl remained from niosomal formulation F4 and niosomal gel formulation G2 stored at 4 ± 1 °C and 37 ± 1 °C.

Time (Days)	Percent of pilocarpine HCl remained ± SD			
	F4		G2	
	4 ± 1^o^C	37 ± 1^o^C	4 ± 1^o^C	37 ± 1^o^C
0	100	100	100	100
7	95.37 ± 1.42	90.36 ± 2.09	96.48 ± 1.23	93.27 ± 3.19
14	90.79 ± 2.32	85.75 ± 1.87	95.35 ± 0.17	90.92 ± 1.17
21	85.54 ± 0.37	80.97 ± 1.51	94.12 ± 0.62	87.19 ± 2.11
28	80.36 ± 1.05	76.45 ± 2.67	86.76 ± 1.25	80.58 ± 1.07

### *Ex vivo* drug permeation study

**Figure 11.**
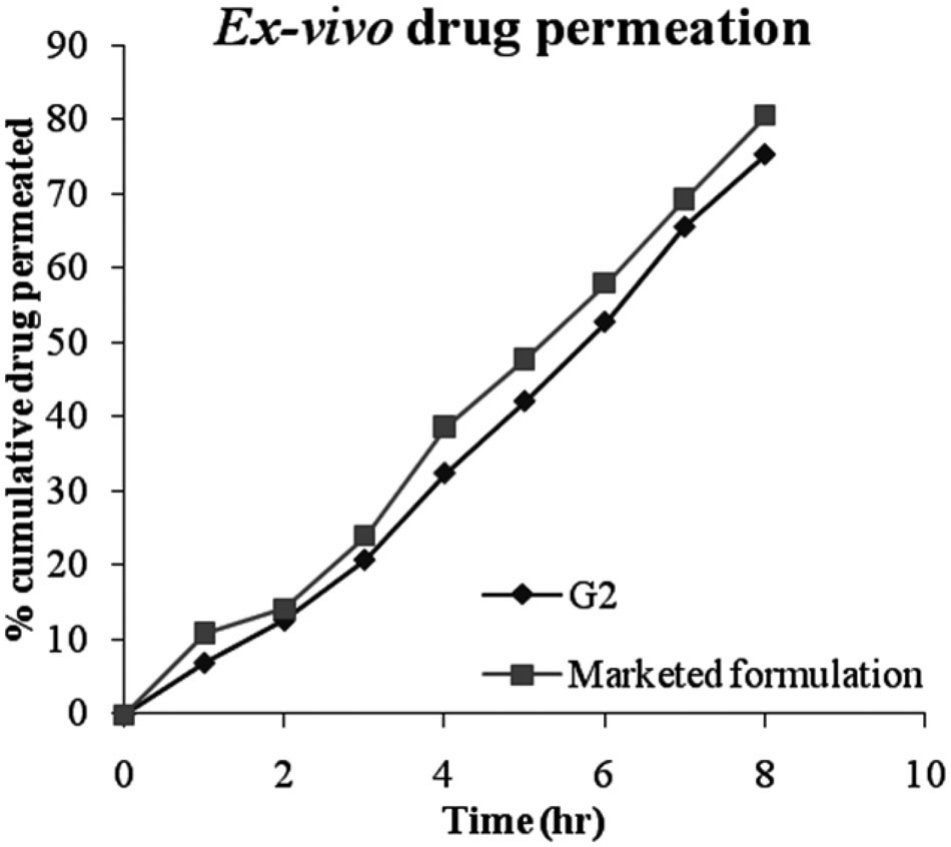
*Ex-vivo* permeation study of selected formulation (G2) and marketed formulation.

The *ex vivo* permeation of pilocarpine HCl from selected formulation G2 and marketed pilocarpine formulation (Pilopine HS gel) through bovine cornea was determined in triplicate and their mean values with standard deviation are shown in Table 9 and the plot of cumulative percent drug permeated as a function of time was shown in Figure 11. The results showed slow and sustained release of drug through the corneal membrane for prolonged period of time in case of niosomal gel formulation (G2) i.e. 75.23 ± 1.25% of drug release in 8 h as compared to marketed formulation i.e. 80.47 ± 0.34% of drug release in 8 h. Faster release of pilocarpine HCl from the marketed gel may be due to the free drug being present in gel structure as compared to niosomal gel in which the drug was entrapped into niosomal vesicular structure. These results were in agreement with Asthana et al., 2016. It has been stated that more hydrophobic span surfactants form more compact niosomes when hydrated in the presence of cholesterol (Bayindir & Yuksel, 2010).

**Table 9.** Results of percent cumulative drug permeated from niosomal gel formulation (G2) and marketed formulation in STF of pH 7.4.

Time (hr)	Average percent cumulative drug permeated (Mean ± SD, n = 3)	
	G2	Marketed formulation
0	0	0
1	06.97 ± 1.23	10.95 ± 0.34
2	12.65 ± 0.86	14.15 ± 0.76
3	20.81 ± 1.98	23.97 ± 0.43
4	32.46 ± 1.17	38.64 ± 1.45
5	42.12 ± 0.24	47.71 ± 1.21
6	52.75 ± 1.65	57.94 ± 0.97
7	65.51 ± 0.67	69.29 ± 0.56
8	75.23 ± 1.25	80.47 ± 0.34

### Ocular irritancy test (draize’s test)

The possibility of eye irritation due to selected niosomal gel formulation (G2) and marketed formulation instillation were evaluated in rabbits. At the point of instillation, rabbit showed slight eye irritation but no signs of redness, inflammation, swelling or increased tear production were observed over the study period for tested formulation. No ophthalmic damage or abnormal clinical signs to the cornea, iris or conjunctivae were visible. This indicated that the nonionic surfactants namely span 60 as well as cholesterol used in the niosome formulations were nonirritant to the eye and could be used safely (Rahimpour & Hamishehkar, 2012). By instillation of marketed Pilopine gel, the rabbit’s eye showed irritation, redness and inflammation at conjunctiva which may be due to its large molecular size, indicating a problem in the conjunctival absorption of drug.

### *In vivo* antiglaucoma activity by measurement of intraocular pressure (IOP)

As shown in Table 10, it was observed that marketed formulation showed a decrease in IOP up to 17.42 ± 1.12 mmHg at the end of 7 h, but then there was an increase in the IOP, which may be due to the elimination of the drug from the site of action. Hence, it was unable to sustain the activity for a long period of time, which calls for frequent administration of the formulation. G2 decreased IOP by 17.98 ± 1.35 mmHg at the end of 9 h. G2 maintain the sustained effect up to 24 h. This control of IOP for prolonged periods may be attributed to the increased corneal residence and sustained drug release of the formulated ophthalmic niosomal gel compared to marketed Pilopine gel. It was also observed that upon administration of pilocarpine HCl ocular niosomal gel, no effect on IOP in the control eye, this may be an indication that no systemic absorption occurred. It was revealed that the sustained effect was maintained for more time in the niosomal gel as compared to the marketed formulation. The marketed Pilopine gel lowered the IOP to minimum (17.42 ± 1.12 mmHg) and afterwards, there was a sudden increase in the IOP upto 40.01 ± 0.43 mmHg in 24 h whereas, niosomal gel lowered the IOP slowly to the minimum and thereafter, a gradual increase in the IOP upto 21.93 ± 1.24 mmHg in 24 h was observed.

**Table 10.** IOP-lowering effects of G2 and marketed formulation after treatment.

Time (hr)	IOP (mmHg) lowering effects of G2 and marketed formulation			
	Group II (G2)		Group III (marketed Pilopine HS gel)	
	R	L	R	L
0	40.47 ± 0.42	40.47 ± 0.04	40.07 ± 0.19	40.07 ± 0.02
2	40.47 ± 0.42	35.12 ± 0.17	40.07 ± 0.19	30.95 ± 1.68
4	40.47 ± 0.42	27.89 ± 0.28	40.07 ± 0.19	27.37 ± 2.32
6	40.47 ± 0.42	23.85 ± 0.31	40.07 ± 0.19	21.69 ± 0.45
7	40.47 ± 0.42	21.20 ± 0.53	40.07 ± 0.19	17.42 ± 1.12
8	40.47 ± 0.42	19.28 ± 0.06	40.07 ± 0.19	20.95 ± 3.21
9	40.47 ± 0.42	17.98 ± 1.35	40.07 ± 0.19	21.69 ± 0.57
10	40.47 ± 0.42	19.54 ± 2.31	40.07 ± 0.19	24.23 ± 1.12
11	40.47 ± 0.42	20.94 ± 0.96	40.07 ± 0.19	28.96 ± 4.96
24	40.47 ± 0.42	21.93 ± 1.24	40.07 ± 0.19	40.01 ± 0.43

**Figure 12.**
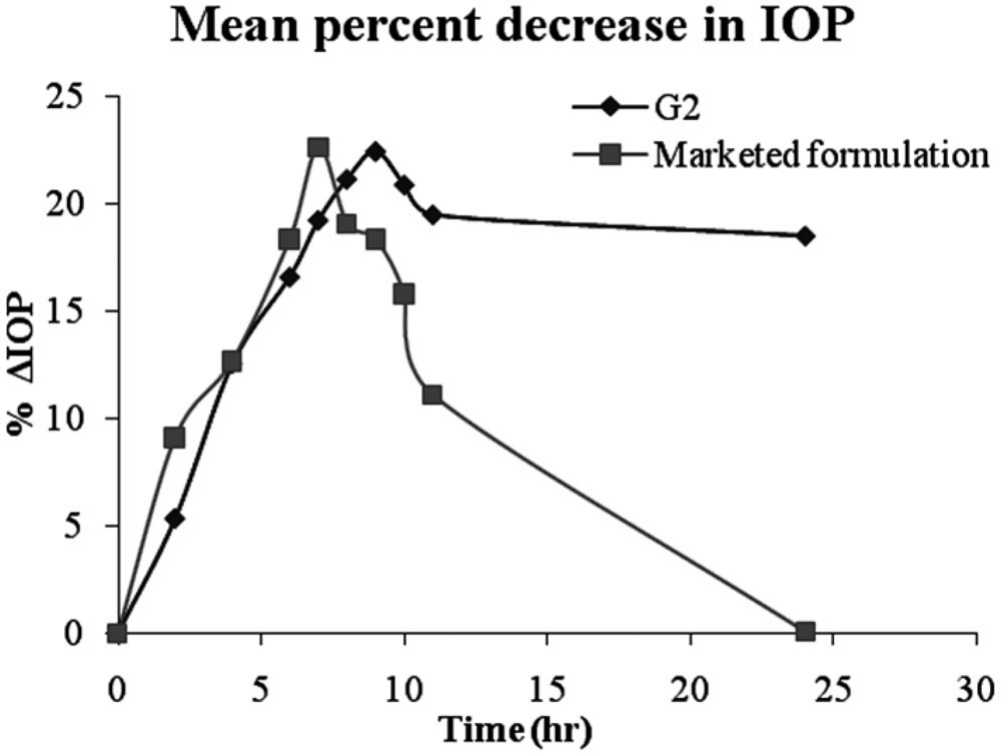
Mean percentage decrease in intraocular pressure (% ΔIOP) versus time.

The mean percentage decrease in IOP after installation of single dose of either niosomal gel formulations (G2) or marketed formulation was calculated and the data was shown in Figure 12. The mean pharmacokinetic parameters for niosomal gel G2 and marketed Pilopine gel was listed in Table 11. The ΔIOP_max_ values for G2 and marketed formulation were 22.49 ± 0.06% and 22.65 ± 1.12% respectively. T_max_ values were 9 h and 7 h respectively. Greater AUC values were observed for G2 (596.99 mmHg.h) compared with marketed Pilopine gel (225.34 mmHg.h). The relative bioavailability for G2 to marketed formulation was 2.64.

**Table 11.** Pharmacokinetic parameters for G2 and marketed Pilopine gel.

Formulations	IOP_max_ (%)	T_max_ (hr)	AUC_0-24 h_ (mmHg.h)
G2	22.49 ± 0.06	9	596.99
Marketed Pilopine gel	22.65 ± 1.12	7	225.34

Higher bioavailability in case of G2 confirmed that the niosomal gel formulation had a prolonged duration of its anti-glaucomatic effect in comparison to the marketed Pilopine gel.

## Conclusion

The results of this study showed that cholesterol content and type of surfactant altered the % EE, vesicle size and release rate from pilocarpine HCl niosomes. Formulation F4 composed of span 60 and cholesterol (1:1) gave the most advantageous entrapment (93.26 ± 1.75%) and release results after 8 hrs (Q8h = 60.35 ± 3.83%) as compared to other compositions. The *in-vitro* release data of gel formulations shows a lower percentage of drug release as compared with niosomes itself. The release data were fitted to an empirical equation to estimate the diffusion parameters, which indicated that the release follows non-Fickian diffusion mechanism. Among all formulations, G2 showed higher bioadhesive strength (2564 ± 0.39 dynes/cm^2^), higher stability while slower drug release in 8 hr due to high gelling capacity. Niosomal gel formulation gave higher AUC than that given by marketed Pilopine gel and increased the bioavailability of pilocarpine hydrochloride by 2.64 times than marketed Pilopine gel. These results suggest that the niosomes containing gels are promising ocular carriers for the controlled delivery of pilocarpine HCl in glaucoma treatment.

### Ethics approval and consent to participate

This protocol was approved by the Institutional Animal Ethical committee of Department of Pharmacy, B.R. Nahata College of Pharmacy, Mandsaur (M.P.)

### Human and animal rights

This article does not contain any studies with human subjects performed by any of the authors; all institutional and national guidelines for the care and use of laboratory animals were followed.
